# Theatre practitioners and organisational adaptive capacity in disaster response

**DOI:** 10.1177/17504589231177833

**Published:** 2023-06-29

**Authors:** Jennifer Klunder-Rosser

**Affiliations:** School of Health and Related Research (ScHARR), University of Sheffield, Sheffield, UK

**Keywords:** Disaster response, Management, Operating theatres, Theatre practitioners, Adaptive capacity, Skill utilisation

## Abstract

Disasters are increasing globally, requiring flexible strategic approaches from healthcare organisations to manage the resultant influx of patients requiring care while also maintaining normal operational services. Theatre practitioners play a key role in disaster response and recovery; however, a lack of appropriate skill utilisation may be reducing overall organisational adaptive capacity and leading to poorer outcomes for organisations, staff and patients. Understanding what skills individual practitioners have, and how they can be deployed to the greatest effect, is a concern for managers to ensure optimal use of resources and to reduce negative impacts of disaster response upon healthcare personnel. This is especially pertinent in the post-COVID healthcare climate where a paucity of operating theatre practitioners and poor workforce planning has led to a lack of surgical capacity at a time when it is most needed.

## Introduction

Disasters, caused by events such as terrorism and climate change, are increasing globally. Public institutions such as healthcare bodies are developing ever-evolving responses to deliver organisational continuity and resilience during response. Healthcare plays an acutely significant role as disasters generally lead to substantial and unpredictable number of people requiring medical intervention (Verheul & Dückers 2020). Resultantly, a priority for healthcare systems is to be resilient; ensuring they have the adaptive capacity (AC) to flexibly respond to an unanticipated demand for provisions, as well as maintain routine operational services (Wiig & O’Hara 2021). Operating theatres (OTs) play a central role in disaster response, providing reactive emergency surgical and anaesthetic care. Registered theatre practitioners (TPs) are highly skilled staff, integral to safe anaesthetic and surgical care, and who form a key component of a flexible OT response (Vogelsang et al 2020). They can be either operating department practitioners (ODPs) or registered nurses (RNs). Undergraduate training pathways for the professions differ, with ODPs undertaking OT-specific training across all three roles (scrub, anaesthetics and recovery). RNs undertake a broader healthcare-wide pathway and may undertake postgraduate training to attain OT-specific competencies such as anaesthetic qualifications and scrub skills (GRIFT 2022). In this paper, the term TPs will be used to refer to both staff groups unless otherwise stated.

Lowes et al (2020) and Vogelsang et al (2020) suggest the TPs skill set is under-researched and institutionally poorly understood. In addition, organisational recovery in the National Health Service (NHS) post-pandemic is undermined by a lack of TPs workforce, reducing organisations’ ability to increase surgical capacity to tackle the extensive backlog of patents awaiting treatment (GRIFT 2022). Retention of staff is a particularly well recognised problem with The King’s Fund (Charles & Ewbank 2021) stating years of poor workforce planning have resulted in a workforce crisis across the NHS, and Mccabe et al (2020) suggesting a lack of nurses is the single biggest barrier to improving organisational recovery. Innovative solutions are required to better attract and retain appropriately skilled TPs. Better understanding the skills and knowledge of TPs to utilise them more effectively during disaster response may go some way to improving their experiences of disaster, therein improving retention, and so organisational AC during disaster recovery. Exploring if there is a difference between the flexibility and utilisation of ODPs’ and RNs’ skill sets in disaster response may also inform future disaster workforce planning and policy. This will allow for more effective utilisation of the TPs skill set during disaster, positively contributing to the overall medical response, and improving overall reactive delivery or surged surgical provision.

## Adaptive capacity (AC)

Organisational resilience in disaster response and recovery does not refer to any one organisational feature, but a number of factors which collectively provide the foundation for flexible response, such as strong leadership and a committed workforce (Lyng et al 2022). AC therefore refers to the ability of organisations to adapt to unpredictability while also ensuring minimal disruption to normal services (Engle 2011, Zhang et al 2018). Rumsey et al (2014) identify four determinants of organisational AC as inter-organisational, intra-organisational, objective and subjective. Intra-organisational determinants, such as leadership and competence, are pertinent to OT management when considering how to best deploy their workforce.

Nevertheless, the adaptive needs of an organisation may conflict with the needs of managers and healthcare professionals as tactical demands can clash with operational pressures and targeted outputs. Organisations need a flexible workforce who can rapidly deploy to most pressurised clinical areas post disaster, a concept known as surge capacity. The need for flexibility in the healthcare workforce is increasingly well understood, with training pathways and policies being developed to encourage this (GRIFT 2022). In addition, the role of psychological safety in staff adaptability is well established, with improved flexibility seen in staff who feel psychological safe in their work environments and leaders managerial practices (Edmondson et al 2016). Despite this, redeployment can be a damaging experience for staff, increasing risk of stress-related illness, mismanagement and negatively affect staff retention, particularly among those who do not feel psychologically safe or well supported by managers (Galura 2020, Willis et al 2021). For TPs, an already scare resource at a high risk of negative occupational mental-health consequences, this risk is further compounded (Cumpsty-Fowler & Saletnik 2021, Esmaeil et al 2022). This creates a danger for managers of a workforce at increased risk of illness and demotivation, undermining the organisation’s ability to recover and adversely affecting their ability to provide surge capability in the time of most pronounced need. Understanding which managerial practices can enhance psychological safety, staff engagement and utilisation may go some way to improving organisation AC in disaster response.

## Staff utilisation and redeployment

Improving TP’s experiences during disaster response and appropriately utilising staff could be a key area of learning for managers to improve inter-departmental and intra-organisational AC. A clinical workforce who possesses the basic knowledge, skills and capabilities to respond adroitly to organisational need is the ideal of disaster response, and redeploying the available workforce to pressurised areas is a vital component to allow hospitals to effectively provide care and manage unpredictable patient flow (Adams & Berry 2012, Veenema et al 2019). However, quality and quantity of staff are independent variables in surge capacity. An oft-neglected priority for disaster management is effectively utilising the specific competencies of individual healthcare professionals, rather than simply increasing the volume of available staff (Faccincani et al 2018, Veenema et al 2016).

The most recent disaster which employed surge workforce methodologies is the COVID-19 pandemic. In the United Kingdom, critical care unit (CCU) capacity was increased by over 70% through a range of redeployment methods, but this came at significant cost to surgical services (Mccabe et al 2020). TPs were prioritised for redeployment to CCU due to the reduced surgical workload and transferability of OT skills to CCU, particularly within anaesthetic and recovery practitioners. This resulted in a 30% reduction in surgical activity to emergency provision only (Melman et al 2021, Price et al 2021, Ryder et al 2021). Subsequently, Hall et al’s (2021) international survey of 173 major trauma centres found 74 centres reported over 50% reduced access to OT and 84.4% reported reduced OT efficiency due to TPs redeployment and increased infection control procedures. By the end of 2021, there were anticipated to be between 3 and 4 million patients awaiting surgery due to COVID-19, and 7.2 million people waiting for elective care as of December 2022 (BMA 2022, Melman et al 2021, Price et al 2021). To overcome these challenges, a significant increase in surgical capacity is required, but a lack of suitable workforce and appropriate management of resources is one of the limiting factors enabling this.

Despite this substantial impact upon surgical capacity, evidence suggests redeployed TPs were not consistently well utilised. Harris & Coopersmith (2021) found that, although 95% of hospitals cancelled elective surgical procedures to redeploy TPs to CCU, less than half utilised their skill set in their deployed roles. This impacted staff wellbeing, with Walker & Gerakios (2021) finding redeployed staff felt frustrated at not being able to employ their skills. This in turn led to feelings of boredom, under appreciation and dissatisfaction, although it is unclear how generalisable these findings are due to limited available qualitative research exploring TP’s experiences. This is suggestive though of a need for managers to reconsider the skill set of individual staff and how they are used in an over-arching healthcare framework. Deployed individuals to skill-specific teams, rather than reactively surging homogeneous staff groups in their entirety to one clinical specialism at the expense of the entire system, may offer an opportunity to improve disaster response workforce policies.

### Possible solutions

Recent research offer possible direction for organisational learning and improved management practices. Oakley et al (2020) redeployed TPs to specialised multidisciplinary teams which maximised their existing skills, such as scrub practitioners to tracheostomy teams. A single study of one hospital’s approach to surge workforce is difficult to generalise more widely, although Lee et al (2020), Vera San Juan et al (2021), Kennedy et al (2022) and Holthof & Luedi (2021) also support this method of task-based teams. A pragmatic hypothesis is that this approach may offer solutions for managers to allow greater exploitation of the available workforce when the resource is limited, while also reducing negative consequences for TPs. However, it is unclear if this practice of skill-specific team occurs consistently, or if this is an isolated approach.

## Conclusion

TPs utilisation is a limited research field, and better understating what are the individual and collective skills practitioners have may allow managers to employ resources more effectively in disaster response and recovery. A body of research looks at organisational response to surge capacity during COVID-19, but little looks at the utilisation of surged staffs’ skills, or what organisational learning could be identified from staff experiences during this type of response. A large body of research examines human factors in healthcare professionals to improve patient safety, generally under ‘normal’ circumstances rather than during disaster (Bjurling-Sjöberg et al 2021, Wiig & O’Hara 2021), or supporting psychological wellbeing during disasters (Heath et al 2020, Kakemam et al 2021). In short, research has looked at how disaster response is organised at a macro level, or how individuals experienced disasters at a micro level, but not how the individual experience impacts upon organisational resilience during disaster response and recovery. Better understanding how the utilisation of TPs skills influences organisational AC is a relatively underexplored area and a priority area for OT managers to develop to improve future resilience.

### Future research

Much of the current literature regarding disaster management focuses either on existing gaps in staffing, the need for surge staffing during a disaster, or the preparedness of Emergency Department and Critical Care staff for disasters. Little empirical research looks at how managers utilise TPs resources, skills or knowledge in disaster response. No research explicitly examines TP’s experiences of disaster, skill utilisation or deployment during disasters to support organisational resilience, and this gap in the research is now the subject of a PhD research project by the author.

## Key points

- Adaptive capacity underpins organisational disaster response.
- Operating theatres play a central role in disaster response.
- Understanding how the skills of theatre practitioners are use in disasters is a key concern for managers.
- Effectively utilising theatre practitioners’ skills during disaster may improve organisational adaptive capacity and recovery after disaster.

## References

[bibr1-17504589231177833] Adams LM Berry D 2012 Who will show up? Estimating ability and willingness of essential hospital personnel to report to work in a disaster Online Journal of Issues in Nursing 17 (2) 8 22686116

[bibr2-17504589231177833] Bjurling-Sjöberg P Göras C Lohela-Karlsson M , et al 2021 Resilient performance in healthcare during the COVID-19 pandemic (ResCOV): Study protocol for a multilevel grounded theory study on adaptations, working conditions, ethics and patient safety BMJ Open 11 (12) e051928 10.1136/bmjopen-2021-051928 PMC8655346 34880017

[bibr3-17504589231177833] BMA 2022 NHS Backlog data analysis Available at: https://www.bma.org.uk/advice-and-support/nhs-delivery-and-workforce/pressures/nhs-backlog-data-analysis (accessed 15 January 2023)

[bibr4-17504589231177833] Charles A Ewbank L 2021 The road to renewal: Five priorities for health and social care Available at: http://www.kingsfund.org.uk/publications/covid-19-road-renewal-health-and-care (accessed 22 October 2021)

[bibr5-17504589231177833] Cumpsty-Fowler C Saletnik L 2021 Fostering a culture of well-being in perioperative nursing: A call to action AORN journal 114 (2) 119–123 34313998 10.1002/aorn.13478

[bibr6-17504589231177833] Edmondson AC Higgins M Singer S Weiner J 2016 Understanding psychological safety in health care and education organizations: A comparative perspective Research in Human Development 13 (1) 65–83

[bibr7-17504589231177833] Engle NL 2011 Adaptive capacity and its assessment Global Environmental Change 21 (2) 647–656

[bibr8-17504589231177833] Esmaeil T Armin Z Saeed B-V Laripour R 2022 Viewpoint of operating room nurses about factors associated with the occupational burnout: A qualitative study Frontiers in Psychology 13 947189 36033007 10.3389/fpsyg.2022.947189 PMC9403988

[bibr9-17504589231177833] Faccincani R Della Corte F Sesana G Stucchi R Weinstein E Ashkenazi I Ingrassia P 2018 Hospital surge capacity during expo 2015 in Milano, Italy Prehospital and Disaster Medicine 33 (5) 459–465 30156181 10.1017/S1049023X18000742

[bibr10-17504589231177833] Galura S 2020 On the frontlines of nursing leadership Nurse Leader 18 (5) 476–480 32837351 10.1016/j.mnl.2020.05.012 PMC7370917

[bibr11-17504589231177833] Getting it Right First Time (GIRFT) 2022 Establishing an effective and resilient workforce for elective surgical hubs Available at: https://www.gettingitrightfirsttime.co.uk/wp-content/uploads/2022/08/Workforce-guidance-for-surgical-hubs-Aug22h.pdf

[bibr12-17504589231177833] Hall AJ Clement ND MacLullich AMJ , et al 2021 IMPACT of COVID-19 on hip fracture services: A global survey by the international multicentre project auditing COVID-19 in trauma & orthopaedics The Surgeon 20 (4) 237–240 34103268 10.1016/j.surge.2021.04.007 PMC8141714

[bibr13-17504589231177833] Harris GH Coopersmith CM 2021 Capacity strain and response during coronavirus disease 2019 Critical Care Medicine 49 (7) 1189–1192 33826585 10.1097/CCM.0000000000005040

[bibr14-17504589231177833] Heath C Sommerfield A Von Ungern-Sternberg BS 2020 Resilience strategies to manage psychological distress among healthcare workers during the COVID-19 pandemic: A narrative review Anaesthesia 75 (10) 1364–1371 32534465 10.1111/anae.15180 PMC7323405

[bibr15-17504589231177833] Holthof N Luedi MM 2021 Considerations for acute care staffing during a pandemic Baillière’s Best Practice and Research in Clinical Anaesthesiology 35 (3) 389–404 10.1016/j.bpa.2020.12.008 PMC7726522 34511227

[bibr16-17504589231177833] Kakemam E Chegini Z Rouhi A Ahmadi F Majidi S 2021 Burnout and its relationship to self-reported quality of patient care and adverse events during COVID-19: A cross-sectional online survey among nurses Journal of Nursing Management 29 (7) 1974–1982 33966312 10.1111/jonm.13359 PMC8237033

[bibr17-17504589231177833] Kennedy E Kennedy P Hernandez J Shakoor K Munyan K. 2022 Understanding redeployment during the COVID-19 pandemic: A qualitative analysis of nurse reported experiences SAGE Open Nursing 8: 237796082211149 10.1177/23779608221114985 PMC9310283 35899038

[bibr18-17504589231177833] Lee CCM Thampi S Lewin B Lim TJD Rippin B Wong WH Agrawal RV 2020 Battling COVID-19: Critical care and peri-operative healthcare resource management strategies in a tertiary academic medical centre in Singapore Anaesthesia 75 (7) 861–871 32267963 10.1111/anae.15074 PMC7262214

[bibr19-17504589231177833] Lowes R Duxbury A Garth A 2020 The evolving roles of operating department practitioners in contemporary healthcare: A service evaluation Journal of Perioperative Practice 30 (3) 46–56 31524064 10.1177/1750458919861913

[bibr20-17504589231177833] Lyng HB Macrae C Guise V Haraldseid-Driftland C Fagerdal B Schibevaag L Wiig S 2022 Capacities for resilience in healthcare; A qualitative study across different healthcare contexts BMC Health Services Research 22 (1) 474 35399088 10.1186/s12913-022-07887-6 PMC8994877

[bibr21-17504589231177833] Mccabe R Schmit N Christen P , et al 2020 Adapting hospital capacity to meet changing demands during the COVID-19 pandemic BMC Medicine 18 (1) 329 33066777 10.1186/s12916-020-01781-w PMC7565725

[bibr22-17504589231177833] Melman GJ Parlikad AK Cameron EAB 2021 Balancing scarce hospital resources during the COVID-19 pandemic using discrete-event simulation Health Care Management Science 24 (2) 356–374 33835338 10.1007/s10729-021-09548-2 PMC8033099

[bibr23-17504589231177833] Oakley C Pascoe C Balthazor D , et al 2020 Assembly line ICU: What the long shops taught us about managing surge capacity for COVID-19 BMJ Open Quality 9 (4) e001117 10.1136/bmjoq-2020-001117 PMC7722360 33277292

[bibr24-17504589231177833] Price J Sheraton T Self R Cook TM 2021 The need for safe, stable and sustainable resumption of planned surgery in an era of COVID-19 Anaesthesia 76 (7) 875–878 33778948 10.1111/anae.15470 PMC8251134

[bibr25-17504589231177833] Rumsey M Fletcher SM Thiessen J , et al 2014 A qualitative examination of the health workforce needs during climate change disaster response in Pacific Island countries Human Resources for Health 12 (1) 9 24521057 10.1186/1478-4491-12-9 PMC3937042

[bibr26-17504589231177833] Ryder M Gallagher P Coughlan B Halligan P Guerin S Connolly M 2021 Nursing and midwifery workforce readiness during a global pandemic: A survey of the experience of one hospital group in the Republic of Ireland Journal of Nursing Management 30 (1) 25–32 34473868 10.1111/jonm.13461 PMC8646494

[bibr27-17504589231177833] Veenema TG Boland F Patton D O’Connor T Moore Z Schneider-Firestone S 2019 Analysis of emergency health care workforce and service readiness for a mass casualty event in the republic of Ireland Disaster Medicine and Public Health Preparedness 13 (2) 243–255 29781406 10.1017/dmp.2018.45

[bibr28-17504589231177833] Veenema TG Griffin A Gable AR , et al 2016 Nurses as leaders in disaster prepardness and response – A call to action Journal of Nursing Scholarship 48 (2) 187–200 26869230 10.1111/jnu.12198

[bibr29-17504589231177833] Vera San Juan N Camilleri M Jeans JP Monkhouse A Chisnall G Vindrola-Padros C 2021 Redeployment and training of healthcare professionals to intensive care during COVID-19: A systematic review Medrxiv Epub ahead of print 23 January DOI: 10.1101/2021.01.21.21250230 PMC8753114 34996785

[bibr30-17504589231177833] Verheul ML Dückers ML 2020 Defining and operationalizing disaster preparedness in hospitals: A systematic literature review Prehospital and Disaster Medicine 35 (1) 61–68 31826788 10.1017/S1049023X19005181

[bibr31-17504589231177833] Vogelsang AC Swenne CL Gustafsson BÅ Brynhildsen KF 2020 Operating theatre nurse specialist competence to ensure patient safety in the operating theatre: A discursive paper Nursing Open 7 (2) 495–502 32089845 10.1002/nop2.424 PMC7024629

[bibr32-17504589231177833] Walker KL Gerakios F 2021 Redeployment during the first wave of the COVID-19 pandemic: Implications for a clinical research workforce British Journal of Nursing 30 (12) 734–741 34170724 10.12968/bjon.2021.30.12.734

[bibr33-17504589231177833] Wiig S O’Hara JK 2021 Resilient and responsive healthcare services and systems: Challenges and opportunities in a changing world BMC Health Services Research 21 (1) 1037 34602063 10.1186/s12913-021-07087-8 PMC8487709

[bibr34-17504589231177833] Willis K Ezer P Lewis S Bismark M Smallwood N 2021 ‘Covid just amplified the cracks of the system’: Working as a frontline health worker during the COVID-19 pandemic International Journal of Environmental Research and Public Health 18 (19) 10178 34639479 10.3390/ijerph181910178 PMC8508504

[bibr35-17504589231177833] Zhang F Welch EW Miao Q 2018 public organization adaptation to extreme events: Mediating role of risk perception Journal of Public Administration Research and Theory 28 (3) 371–387

